# A Source-Aware and Physically Interpretable Data-Driven Framework for Predicting Semi-Circular Bending (SCB) Fracture Energy of Fiber-Reinforced Asphalt Mixtures

**DOI:** 10.3390/ma19102172

**Published:** 2026-05-21

**Authors:** Wu Zeng, Zhiyou Ge, Lingyan Shan, Huanwang Liao, Qing Xiao, Xunqian Xu

**Affiliations:** 1 Jiangxi Communications Investment Group Co., Ltd., Nanchang 330108, China; zw030717@163.com (W.Z.); lhwmdpi@163.com (H.L.); 2 Institute of Artificial Intelligence, Nantong University, No. 9 Seyuan Road, Nantong 226019, China; gzyntu@163.com (Z.G.); xunqian_xu@ntu.edu.cn (X.X.); 3 Research Institute of Highway, Ministry of Transport, Beijing 100088, China

**Keywords:** fiber-reinforced asphalt mixture, semi-circular bending (SCB) test, fracture energy, source-aware validation, SHapley Additive exPlanations (SHAP) analysis, fracture mechanics, mixture design

## Abstract

Fiber-reinforced asphalt mixtures improve cracking resistance through fiber bridging, pull-out, and crack-path deflection, but their semi-circular bending (SCB) fracture energy is affected by coupled mixture, testing, and fiber-related variables. This study developed a source-aware and physically interpretable data-driven framework for predicting SCB fracture energy using a literature-derived database containing 261 valid sample-level records from nine source groups. The database was constructed through semantic extraction, unit normalization, rule-based checking, manual verification, and source identifier (SourceID) tracking. Optimum asphalt content, air voids, test temperature, loading rate, fiber dosage, fiber length, diameter, elastic modulus, and tensile strength were used as input variables. Under sample-wise testing, the selected model achieved a coefficient of determination (R^2^) of 0.89, a root mean square error (RMSE) of 0.0470 kJ/m^2^, and a mean absolute error (MAE) of 0.0247 kJ/m^2^ for the full dataset, while the fiber-containing subset achieved R^2^ = 0.94, RMSE = 0.0194 kJ/m^2^, and MAE = 0.0103 kJ/m^2^. Source-aware validation showed higher prediction errors, indicating that cross-source generalization remains more challenging than internal sample-wise prediction. SHapley Additive exPlanations (SHAP) analysis identified temperature, fiber dosage, and fiber mechanical descriptors as dominant contributors, consistent with temperature-dependent viscoelasticity, fiber bridging, and pull-out mechanisms. The dosage–response analysis was restricted to the observed fiber-dosage range of 0–0.678%, providing a bounded screening tool rather than an extrapolative design equation.

## 1. Introduction

Asphalt pavements are vulnerable to low-temperature cracking, fatigue cracking, and reflective cracking under the long-term coupling of thermal cycling and traffic loading. Once cracks form, they often propagate rapidly, are expensive to repair, and substantially reduce service performance. Because crack propagation is governed by the competition among energy input, damage evolution, and energy dissipation, peak strength alone cannot adequately characterize the full anti-cracking capacity of a mixture. Fracture energy (Gf), defined as the energy consumed per unit of newly created crack surface, provides a more direct measure of the comprehensive energy dissipation from crack initiation to propagation and has become an important indicator in balanced mix design and cracking-resistance evaluation [1,2,3].

The semi-circular bending (SCB) test is widely used to evaluate fracture-related indices because specimen preparation and loading are relatively simple and stable load–displacement responses can be obtained [2,3]. In fiber-reinforced asphalt mixtures, fibers alter the crack path and the dissipation pattern through bridging, pull-out, crack deflection or branching, and stress redistribution at the crack tip. As a consequence, fracture energy is more sensitive than a single strength index for capturing the toughening contribution of fibers. At the same time, fiber dosage and fiber geometry and mechanical properties interact strongly with volumetric parameters, temperature, and loading rate, which leads to pronounced nonlinear and interactive responses [4,5,6,7,8].

Although many experimental studies have confirmed that fibers can improve cracking resistance, several engineering questions remain unresolved. First, fracture energy is jointly controlled by temperature, volumetric state, loading regime, and fiber-related dissipation mechanisms; therefore, empirical relationships derived from limited test points are often local and difficult to generalize. Second, published SCB datasets are heterogeneous because specimen dimensions, notch configurations, temperature protocols, loading rates, and reported metrics vary across studies. Third, most existing studies provide pointwise comparisons under one fiber type, one dosage, and one temperature, which is insufficient for practical design questions such as whether performance improvements remain stable within the data-supported dosage domain, whether diminishing returns or thresholds exist at low fiber dosages, and how mean gain and uncertainty should be considered simultaneously under different background mixture designs [4,5,6,7,8].

Previous studies have explored machine learning approaches for predicting fracture-related properties of asphalt mixtures, including low-temperature fracture energy and cracking indices. These studies demonstrated the feasibility of models such as artificial neural networks (ANNs), gene expression programming (GEP), augmented full quadratic models (AFQMs), and self-validated ensemble modeling (SVEM) for asphalt-mixture fracture prediction [9]. Cohesive-zone and mesostructure-based numerical studies have also shown that fracture energy is closely related to traction–separation behavior, aggregate–mastic interaction, and temperature-dependent fracture processes [10,11]. However, most existing studies were not specifically designed for fiber-reinforced SCB fracture-energy data, nor did they systematically address source-level heterogeneity in literature-derived datasets. Therefore, the present study does not claim novelty in the machine learning algorithms themselves; rather, its contribution lies in constructing a source-tracked fiber-reinforced SCB fracture-energy database, evaluating source-aware generalization, and linking statistical model interpretation with fiber-toughening mechanisms [9,10,11].

To address these issues, this study uses SCB fracture energy as the sole output and constructs a unified input system using optimum asphalt content (OAC), air voids (AV), test temperature (Temp), loading rate (Rate), fiber dosage (Fiber%), fiber length (L), fiber diameter (D), elastic modulus (E), and tensile strength (TS). A complete workflow is then established that combines distribution statistics, Kendall correlation analysis, an 80/20 train–test split, five-fold cross-validation, multi-model comparison, SHAP-based interpretability, and bounded dosage scanning [12,13,14,15,16,17,18,19,20,21,22,23,24]. Compared with conventional point prediction, the work emphasizes an engineering-oriented representation in which dosage–response curve clusters are used to characterize both the gain trend and the dispersion risk across multiple mixture backgrounds.

The main contributions of this study are as follows. First, a source-tracked SCB fracture-energy database for fiber-reinforced asphalt mixtures was constructed from heterogeneous published studies, with each record assigned a sample-level RecordID and each literature source assigned a SourceID. Second, the study explicitly distinguishes sample-wise prediction accuracy from source-aware generalization, thereby addressing the risk of source-level dependence in literature-derived datasets. Third, SHAP-based statistical attribution is interpreted together with fracture-mechanics concepts, including fiber bridging, pull-out, crack deflection, and temperature-dependent viscoelastic response. Fourth, the dosage–response curve cluster analysis is reformulated as a bounded engineering screening tool within the observed fiber-dosage range rather than as an extrapolative design equation.

## 2. Materials and Methods

### 2.1. Literature-Derived Database Construction and Variable System

Because relevant SCB fracture-energy data are dispersed across published studies and are reported with heterogeneous terminology, tables, figure captions, and narrative descriptions, the database was constructed through a literature-to-table workflow rather than from a single experimental campaign. Peer-reviewed studies were considered eligible when they reported SCB fracture energy, or sufficient load–displacement/area information to identify the fracture-energy result, together with clearly traceable mixture and test-condition descriptors. Records were excluded when the SCB indicator could not be aligned with fracture energy, when key variables or units were ambiguous, when duplicate experimental conditions were repeated across text and tables, or when the information could not be normalized after manual checking. Each unique mixture–temperature–loading condition was treated as one sample. After extraction, cleaning, deduplication, and consistency checking, 261 valid samples were retained for modeling. The final modeling matrix was stored in the data_gf_scb sheet and contained 261 sample-level records. Each record was assigned a unique record identifier (RecordID), and each literature source was assigned a source identifier (SourceID). The final dataset consisted of nine source groups, including S5, S11, S7, Marasteanu2007_ROSA38925, AlbertaInnovates2021_Hesp, S6, S8, S10, and S9. Synthetic or virtual rows were removed before modeling. Zero-fiber reference mixtures were preserved in the full dataset, whereas records with Fiber% > 0 formed the fiber-containing subset used for mechanism-oriented interpretation.

**Figure 1:**
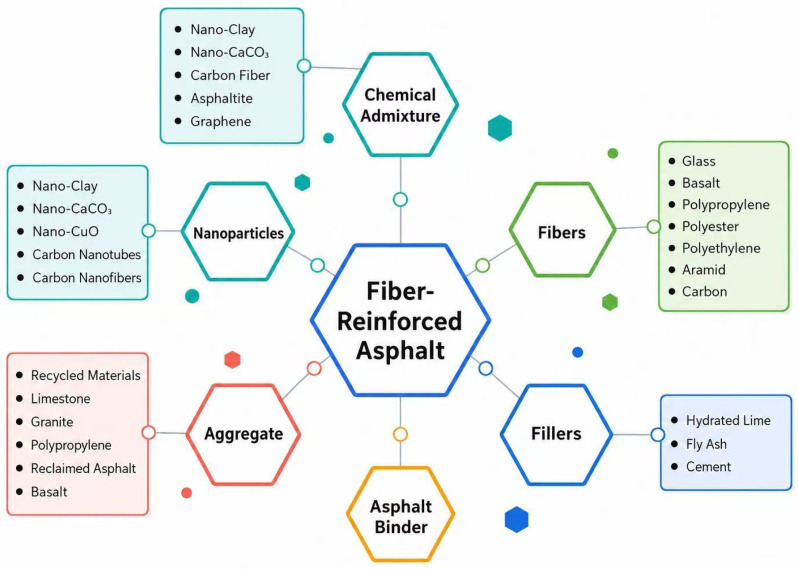
Schematic diagram of the material composition and variable system for fiber-reinforced asphalt mixtures.

To improve consistency across heterogeneous sources, a three-stage processing route was adopted: semantic extraction, rule-based normalization, and manual verification. Synonymous fields such as optimum binder content and optimum asphalt content were mapped to OAC, while fiber-dosage expressions were unified as Fiber%. Units were standardized to %, mm, μm, GPa, MPa, and kJ/m2. Missing fiber descriptors were encoded as zero only for intentionally fiber-free reference samples; otherwise incomplete records were not used for model fitting. Figure 1 presents the conceptual framework of the material system and the variable hierarchy adopted in this study. The asphalt binder, aggregate skeleton, and filler mastic constitute the matrix system, whereas fibers serve as the reinforcing phase; additional modifiers such as nano-materials or chemical additives may act as synergistic agents. For prediction purposes, OAC and AV were treated as volumetric variables describing the binder–skeleton state, temperature and loading rate were regarded as environmental and testing variables, and Fiber%, L, D, E, and TS were taken as mechanism-related variables controlling bridging and pull-out behavior. The definitions, units, encoding methods, and physical meanings of the input and output variables are summarized in Table 1. The complete source-tracked modeling matrix is provided in Supplementary Table S1, and the SourceID mapping and sample-count summary are provided in Supplementary Table S2. The source-level distribution of the final modeling dataset is provided in Appendix A.

### 2.2. Fracture-Mechanics Basis of SCB Fracture Energy

SCB fracture energy represents the work required to create a unit fracture surface during crack initiation and propagation. It can be expressed as:

(1) $$G_{f}\;=\;W_{f}\;/\;A_{lig\;}$$

where W_f_ is the work of fracture obtained from the area under the load–displacement curve, and A_lig is the effective ligament area. From a cohesive-fracture perspective, fracture energy can also be related to the area under a traction–separation law:

(2) $$G_{f}=\int_{0}^{\delta_{c}}T(\delta )d\delta$$

where T(δ) is the cohesive traction and δ_c is the critical crack-opening displacement [10,11].

In fiber-reinforced asphalt mixtures, fibers may increase Gf through crack bridging, interfacial debonding, pull-out energy dissipation, crack deflection, and redistribution of stress near the crack tip. Temperature affects the viscoelastic state of the binder matrix and therefore changes the balance between brittle fracture and ductile energy dissipation. Accordingly, the selected variables in this study are not only statistical predictors but also descriptors related to the physical mechanisms governing SCB fracture energy.

### 2.3. Data Distribution and Descriptive Statistics

**Figure 2:**
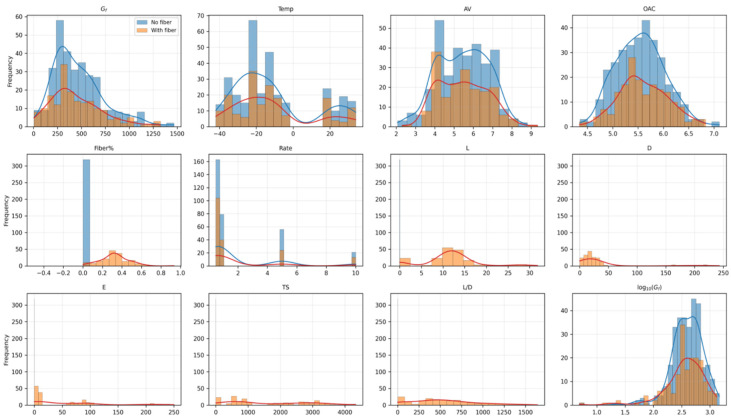
Distribution comparison between the full dataset and the fiber-containing subset. The blue and red lines represent the full dataset and the fiber-containing subset, respectively.

Before model training, the data distribution must be examined to determine whether the variable ranges are representative, whether skewness or long-tail behavior is present, and whether the fiber-containing and fiber-free samples differ systematically. Figure 2 therefore compares the distributions of the output and the input variables between the full dataset and the fiber-containing subset. The purpose of this figure is not limited to graphical description; it also supports subsequent methodological choices, such as the use of robust correlation measures, the interpretation of possible interaction effects, and the identification of the data-supported domain for dosage scanning.

Table 2 summarizes the descriptive statistics calculated from the 261 records in the data_gf_scb modeling matrix. Figure 2 was plotted from the same dataset to compare the full dataset and the fiber-containing subset. Therefore, Table 2 and Figure 2 were obtained directly from the cleaned and source-tracked modeling database rather than from simulated or virtual records. The values show that the database covers typical engineering ranges in terms of mixture composition, temperature, and loading regime. The concentration of Fiber% at low dosage levels also reflects common engineering practice, in which modest dosage levels are often preferred to balance toughening benefit, workability, and cost. Importantly, the observed Fiber% maximum is 0.678%; therefore, interpretations of model behavior above this level must be treated as exploratory rather than strictly validated by direct data coverage.

### 2.4. Correlation Analysis

**Figure 3:**
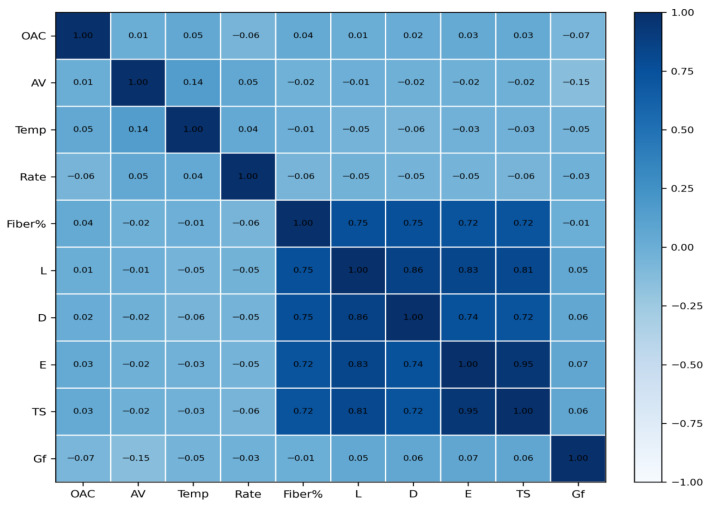
Kendall correlation matrix of the full dataset.

**Figure 4:**
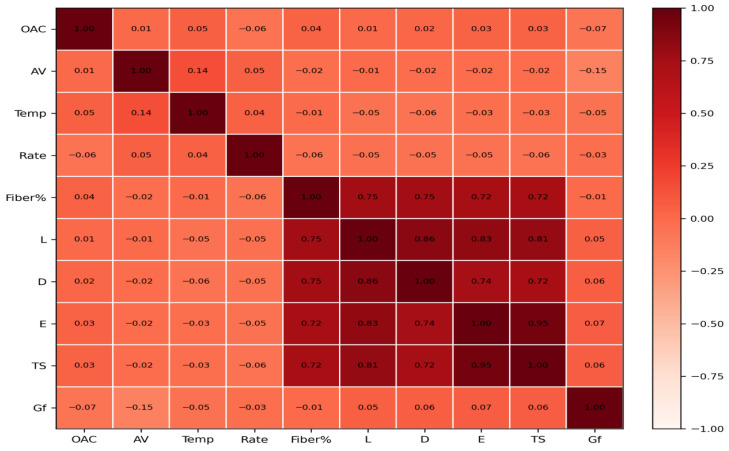
Kendall correlation matrix of the fiber-containing subset.

To explore the overall association structure among the variables, Kendall correlation coefficients were calculated for both the full dataset (Figure 3) and the fiber-containing subset (Figure 4). Kendall correlation is suitable for nonnormal engineering data and is more robust than Pearson correlation when outliers or skewed distributions are present. In this study, the correlation matrices are used as a pre-modeling structural map rather than as evidence of causality. It should be emphasized that Kendall correlation analysis was not used as a predictive model. It was used only as a nonparametric statistical tool to examine monotonic associations before model training. The color scale in Figure 3 and Figure 4 represents Kendall’s τ coefficient, where warmer colors indicate positive monotonic association, cooler colors indicate negative monotonic association, and values close to zero indicate weak monotonic association.

The comparison between the full sample and the fiber-containing subset is particularly important because the mechanism controlling fracture energy changes when fibers participate in crack bridging and pull-out. The subset analysis therefore helps reveal whether fiber-related variables gain explanatory importance once the response is dominated by reinforcement effects.

The color scale represents Kendall’s τ coefficient; red indicates positive association, blue indicates negative association, and colors near white indicate weak association.

### 2.5. Sample-Wise and Source-Aware Validation Strategy

The model was first evaluated using a sample-wise 80/20 train–test split and five-fold cross-validation, which are commonly used for internal predictive assessment. However, because the database was derived from multiple literature sources, samples from the same source may share similar specimen geometry, material system, test protocol, or reporting format. To examine the risk of source-level dependence, the retained SourceID field was used to conduct source-aware diagnostic validation. In this validation, records from the same literature source were kept within the same group. Three source-aware strategies were considered: GroupKFold validation, repeated group shuffle splitting, and leave-one-source-out validation. These tests were not used to replace the sample-wise performance metrics but to evaluate whether the trained model could generalize across literature sources.

**Figure 5:**
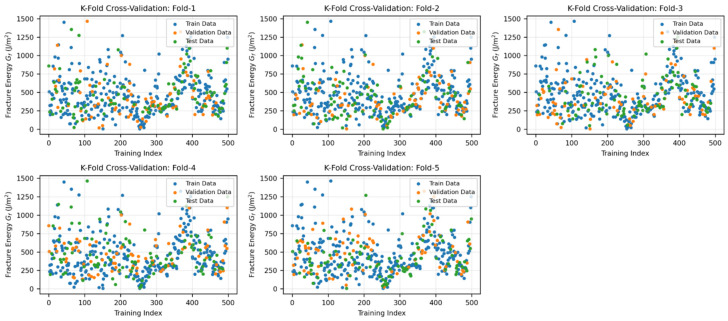
Five-fold cross-validation distribution of the full dataset.

**Figure 6:**
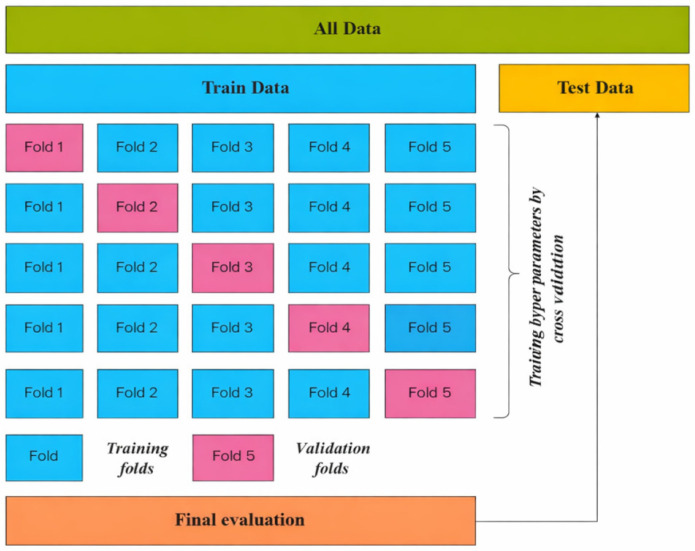
Five-fold cross-validation workflow of the full dataset.

**Figure 7:**
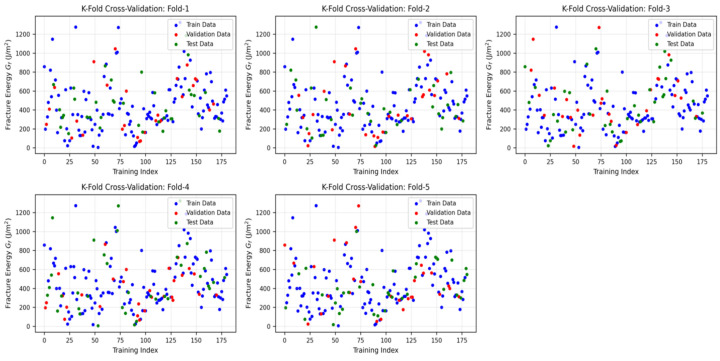
Five-fold cross-validation distribution of the fiber-containing subset.

Figure 5 displays the distribution of the full dataset across the five sample-wise fold. Figure 6 illustrates the workflow used to avoid information leakage between model selection and final testing, while Figure 7 shows the corresponding fold distribution of the fiber-containing subset.

### 2.6. Model System, Evaluation Metrics, and Interpretation Strategy

Several machine learning models were compared to establish the prediction framework, including categorical boosting (CatBoost), extreme gradient boosting (XGBoost), gradient boosting machines (GBMs), extremely randomized trees (Extra Trees), and a multi-layer perceptron (MLP). The representative hyperparameters are listed in Table 3. Model performance was evaluated on both training and testing sets using R^2^, MSE, RMSE, and MAE. To keep the target magnitude numerically convenient during training and error reporting, fracture energy in kJ/m^2^ was scaled as:

(3) $$y^{*}\;=\;100\;\times \;G_{f}$$

Therefore, the coefficient of determination (R^2^) is unaffected by scaling, whereas the reported mean squared error (MSE), root mean square error (RMSE), and mean absolute error (MAE) correspond to the scaled target. The conversion back to physical units is:

(4) $${\mathrm{MSE}}_{\mathrm{Gf}}={\mathrm{MSE}}_{y^{*}}/10^{4}$$

(5) $${\mathrm{RMSE}}_{\mathrm{Gf}}={\mathrm{RMSE}}_{y^{*}}/100$$

(6) $${\mathrm{MAE}}_{\mathrm{Gf}}={\mathrm{MAE}}_{y^{*}}/100$$

In addition to these scalar metrics, error sequences, error histograms, and the observed–predicted relationship with a ±10% error band were used to judge engineering applicability. To enhance model robustness, hyperparameters were tuned empirically, and feature normalization and data cleaning were applied to reduce heterogeneity across literature sources.

To interpret the selected models, SHAP analysis was conducted at three levels: mean absolute contribution ranking, beeswarm plots showing both magnitude and direction, and heatmaps showing contribution structure along the sample dimension. Finally, Fiber% was scanned within the observed dosage range of 0–0.678% under multiple background mixture designs to form dosage–response curve clusters and an average trend line for scheme comparison. Because the literature database is concentrated in the low-dosage domain and the maximum observed Fiber% is 0.678% (Table 2), the dosage–response analysis is interpreted as data-supported interpolation within this interval rather than as an extrapolation-based design recommendation.

**Figure 8:**
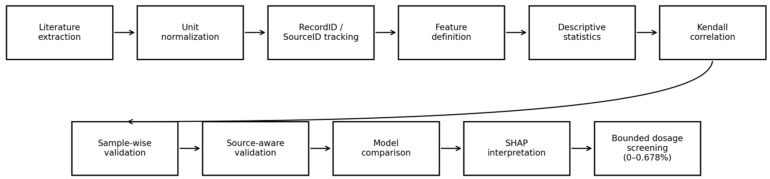
Overall workflow of the proposed source-aware and physically interpretable data-driven framework.

Figure 8 summarizes the overall workflow of the proposed framework, including literature data extraction, unit normalization, source identifier (SourceID) tracking, descriptive analysis, sample-wise and source-aware validation, model comparison, SHAP interpretation, and bounded dosage–response screening. This workflow was designed to ensure that data curation, prediction, interpretation, and engineering screening were connected within a reproducible analysis route.

## 3. Results

### 3.1. Overall Workflow and Distribution Characteristics

Figure 2 confirms that the dataset spans practical engineering conditions and that several variables exhibit concentrated ranges rather than uniform coverage. In particular, the fiber dosage is heavily distributed in the low-dosage region, which is consistent with practice-oriented research strategies. Some fiber mechanical properties also show discrete or grouped distributions, indicating that product-bound correlations may exist among geometry and mechanical parameters. This pattern also explains why the screening-oriented dosage scan must be interpreted more confidently inside the observed low-dosage region than outside it.

The descriptive statistics in Table 2 further show that Gf ranges from 0.005 to 1.324 kJ/m^2^, with a mean of 0.448 kJ/m^2^. The broad temperature range from −42 to 34 °C and the variability in OAC and AV provide a reasonable basis for studying coupled effects between material design and testing conditions. By contrast, the Fiber% distribution is much narrower, with a database maximum of 0.678%, which sets the evidence-supported upper bound for direct interpretation of the dosage scan.

### 3.2. Correlation Structure of the Full Dataset and Fiber Subset

The correlation matrices reveal that the monotonic association between Gf and individual variables is generally weak in the full dataset, which is consistent with the expectation that fracture energy is governed mainly by nonlinear and interactive effects. By contrast, strong internal correlations appear among several fiber descriptors, indicating product-level coupling between geometry and mechanical grade.

In the fiber-containing subset, the structure becomes more informative for mechanism analysis. Fiber-related variables are more clearly linked with the response pattern, supporting the use of a separate subset analysis for interpretability and dosage-oriented decision making.

### 3.3. Multi-Model Performance Comparison

**Figure 9:**
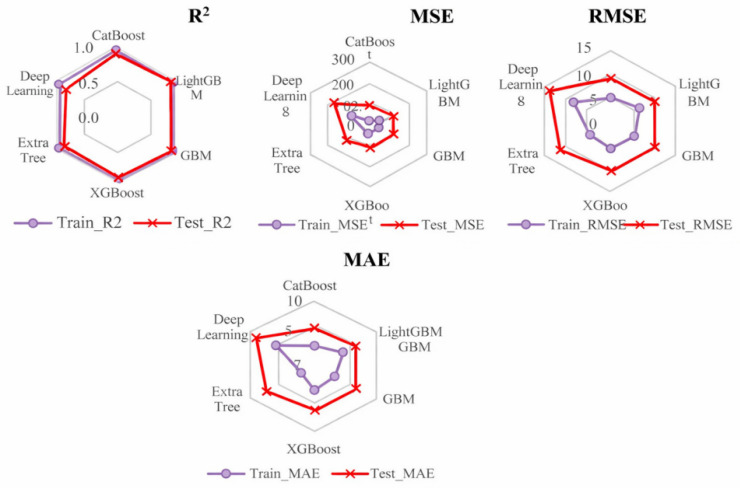
Radar chart of multi-model performance on the full dataset.

**Figure 10:**
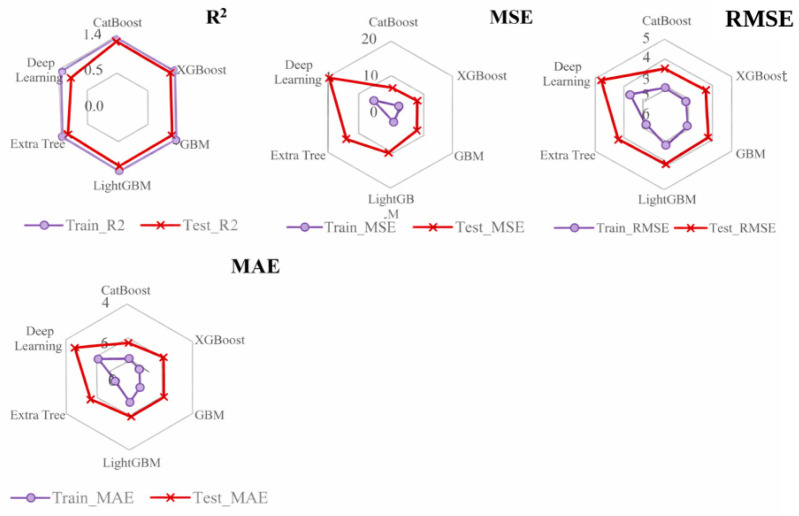
Radar chart of multi-model performance on the fiber-containing subset.

Figure 9 and Figure 10 compare the training and testing performance of the candidate models for the full dataset and the fiber-containing subset, respectively. Using the scaled target defined in Equation (2), the selected model achieved R^2^ = 0.89, MSE = 22.06, RMSE = 4.70, and MAE = 2.47 on the full test set. These values correspond to 0.002206 (kJ/m^2^)^2^, 0.0470 kJ/m^2^, and 0.0247 kJ/m^2^ in physical units. For the fiber-containing subset, the corresponding testing metrics improved to R^2^ = 0.94, MSE = 3.75, RMSE = 1.94, and MAE = 1.03 on the scaled target, equivalent to 0.000375 (kJ/m^2^)^2^, 0.0194 kJ/m^2^, and 0.0103 kJ/m^2^ in physical units.

These sample-wise results indicate that the selected variables can reproduce internal patterns within the compiled dataset. Table 4 also shows that, within the fiber-containing subset, XGBoost yields the highest mean cross-validated R^2^ among the compared models, whereas the MLP performs poorly under the present data volume. However, when records are separated by literature source, the predictive performance decreases substantially, indicating that part of the sample-wise accuracy may be associated with source-specific experimental conditions, material systems, or reporting patterns. Therefore, the model is more appropriate for preliminary screening and factor identification than for direct source-independent design prediction.

### 3.4. Source-Aware Validation Diagnosis

Although the sample-wise test results showed high predictive accuracy, the source-aware diagnostic validation revealed a clear decrease in cross-source generalization. The GroupKFold validation produced a mean R2 of −0.887, RMSE of 0.273 kJ/m2, and MAE of 0.225 kJ/m2. The leave-one-source-out validation produced a mean R2 of −5.417, RMSE of 0.344 kJ/m2, and MAE of 0.311 kJ/m2. Repeated group shuffle splitting yielded a mean R2 of −2.110, RMSE of 0.323 kJ/m2, and MAE of 0.278 kJ/m2. These results indicate that the sample-wise model captures internal patterns within the compiled database, whereas prediction across independent literature sources remains challenging. Therefore, the high sample-wise R2 should not be interpreted as definitive evidence of source-independent generalization. The detailed source-aware validation results are summarized in Table 5, and the corresponding validation script and output are provided as Code S1 and Output S1, respectively.

### 3.5. Error Diagnostics and Observed–Predicted Relationship

**Figure 11:**
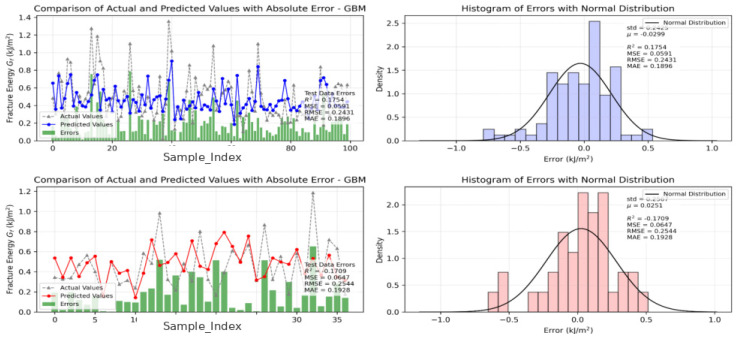
Prediction sequence, absolute error, and error distribution.

Figure 11 combines prediction sequence comparison, absolute error bars, and error histograms. This integrated view is useful because average metrics alone cannot show whether the model exhibits local bursts of error, systematic overestimation or underestimation, or long-tail behavior under extreme conditions. The full-sample error distribution is approximately centered around zero, with a reported mean of about −0.03 and a standard deviation of about 0.24 on the normalized error scale, suggesting no strong global bias but still some uncertainty under extreme cases.

**Figure 12:**
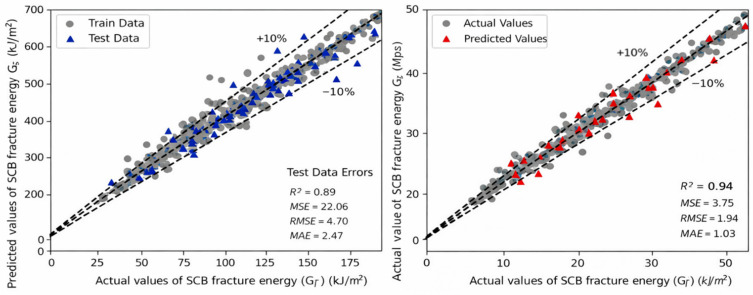
Observed–predicted relationship with the ±10% error band.

Figure 12 further evaluates the engineering usefulness of the selected model through the observed–predicted relationship and the ±10% error band. Most points are distributed close to the diagonal, although the scatter increases in the high-response region, implying that additional high-performance samples would still be beneficial for future calibration. This is another reason why the dosage scan is most credible inside the low-dosage, data-supported region.

### 3.6. SHAP-Based Interpretability Analysis

**Figure 13:**
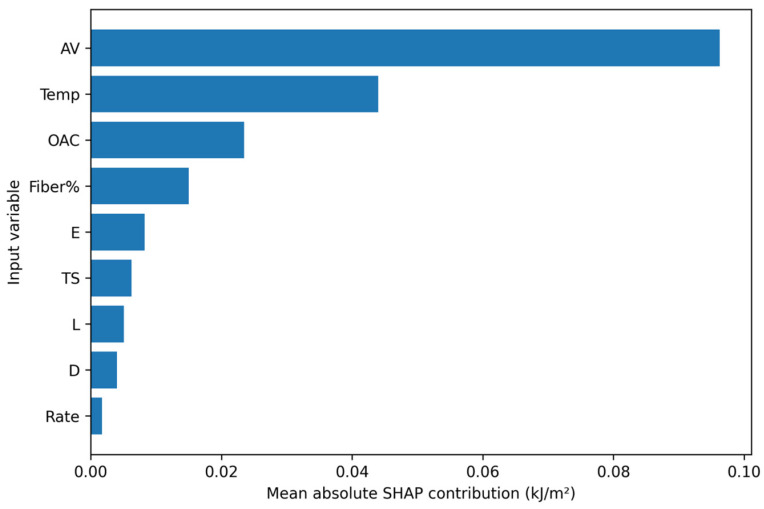
Ranking of mean absolute SHAP contributions.

**Figure 14:**
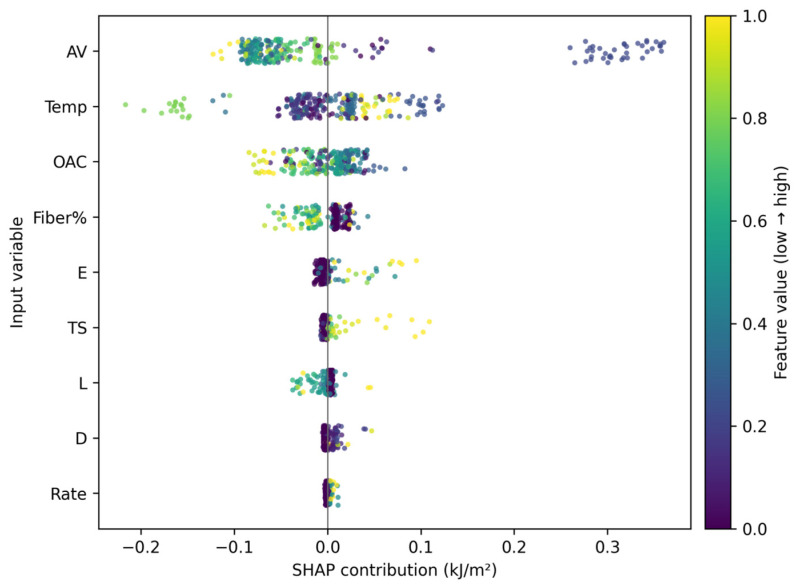
SHAP beeswarm plot.

**Figure 15:**
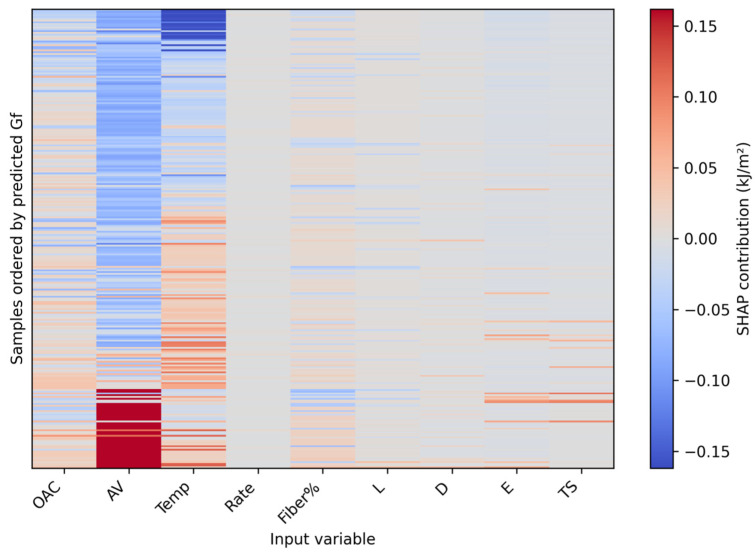
SHAP heatmap of the selected model.

The SHAP results in Figure 13, Figure 14 and Figure 15 provide a coherent interpretation chain from variable ranking to directional effects and finally to sample-wise contribution structure. In the full dataset, temperature, Fiber%, AV, and fiber geometry and mechanical properties all contribute substantially to model output. In the fiber-containing subset, fiber-related variables such as Fiber%, L/D, E, and TS become even more important, which agrees with the mechanistic expectation that reinforcement behavior dominates once fibers actively participate in crack bridging and pull-out.

It should be noted that SHAP values provide statistical attribution within the trained model and should not be interpreted as direct physical causation. The observed sign changes in SHAP values may arise from nonlinear interactions among temperature, fiber dosage, fiber geometry, and mixture volumetrics, but they may also reflect source heterogeneity and uneven data coverage. Therefore, the SHAP results were interpreted only when they were consistent with known fracture mechanisms, such as temperature-dependent binder viscoelasticity, fiber bridging, pull-out resistance, and crack-path deflection.

Each row represents one sample, each column represents one input variable, and the color bar represents the SHAP contribution to the predicted SCB fracture energy. Samples were ordered according to the predicted Gf values to show contribution changes across the response domain.

### 3.7. Dosage Scanning and Fiber Scheme Comparison

**Figure 16:**
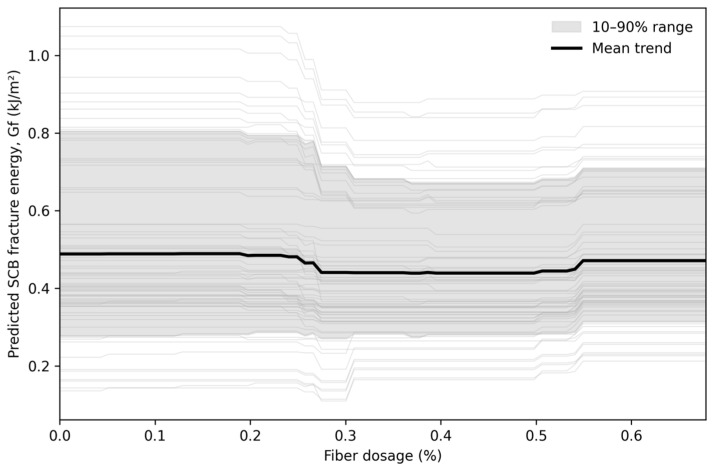
Dosage response curve clusters within the observed fiber-dosage range of 0–0.678%. Different colored curves represent different background mixture conditions, and the mean curve represents the average dosage–response trend.

Figure 16 presents dosage–response curve clusters obtained by scanning Fiber% within the observed data-supported range from 0 to 0.678%. The individual curves represent different background mixture conditions, and the mean trend summarizes the average response. This bounded scanning strategy avoids unsupported extrapolation beyond the maximum fiber dosage available in the compiled database.

Within the observed dosage range, the model does not indicate a universally monotonic dosage–response relationship. Instead, the predicted response shows weakly nonlinear and background-dependent variation, suggesting that the effect of fiber dosage is strongly modulated by temperature, volumetric state, loading rate, and fiber descriptors. A response cluster with limited dispersion may be considered more robust, whereas wide dispersion indicates stronger sensitivity to background mixture and testing conditions. Therefore, the dosage–response cluster should be regarded as a preliminary screening tool for low-dosage fiber design rather than a validated optimization equation.

## 4. Discussion

### 4.1. Physical Interpretation of Dominant Variables

The dominance of temperature is physically reasonable because asphalt mixtures exhibit strong temperature-dependent viscoelastic behavior. At low temperatures, the binder matrix becomes stiffer and more brittle, leading to reduced crack-tip relaxation capacity. At higher temperatures, increased ductility may enhance energy dissipation but may also reduce load-carrying capacity. Fiber-related variables influence fracture energy through bridging and pull-out mechanisms. Longer fibers may provide a longer pull-out path and stronger bridging capacity, whereas diameter, modulus, and tensile strength affect interfacial stress transfer and rupture resistance. However, these descriptors are often coupled at the product level, which explains why their effects should be interpreted jointly rather than independently.

### 4.2. Source Heterogeneity and Generalization

The source-aware validation results reveal that cross-source generalization is substantially more difficult than sample-wise prediction. This finding is important because literature-derived datasets inevitably combine different specimen geometries, notch configurations, mixture designs, loading protocols, and reporting standards. Therefore, the high sample-wise R^2^ values mainly demonstrate internal consistency within the compiled database, whereas the negative source-aware R^2^ values indicate that the current model should not be used as a universal design equation without source-specific calibration. This result does not invalidate the framework; instead, it defines its appropriate use as a preliminary screening and interpretation tool.

### 4.3. Engineering Implications and Limitations

The main engineering implication of this study is that the proposed framework can help identify dominant variables and screen low-dosage fiber schemes before conducting extensive laboratory tests. However, several limitations remain. First, the database is literature-derived, and residual heterogeneity in specimen geometry, notch configuration, mixture design, and reporting format cannot be completely eliminated. Second, the source-aware validation results show that cross-source prediction remains weak, indicating that future datasets should include more balanced source groups and standardized testing protocols. Third, the observed fiber-dosage range is limited to 0.678%, so the dosage–response analysis should not be extrapolated to high fiber contents. Fourth, SHAP analysis provides statistical attribution rather than physical causation, and its interpretation must be constrained by fracture-mechanics knowledge.

## 5. Conclusions

(1) A source-aware and physically interpretable data-driven framework was developed for predicting SCB fracture energy in fiber-reinforced asphalt mixtures using OAC, AV, Temp, Rate, Fiber%, L, D, E, and TS as inputs. The framework integrates literature-derived data extraction, source-ID tracking, field normalization, distribution analysis, Kendall correlation, sample-wise validation, source-aware validation, multi-model comparison, SHAP interpretation, and bounded dosage scanning.
(2) Based on the scaled target defined in Equation (2), the full-sample test performance reached R^2^ = 0.89, MSE = 22.06, RMSE = 4.70, and MAE = 2.47, while the fiber-containing subset achieved R^2^ = 0.94, MSE = 3.75, RMSE = 1.94, and MAE = 1.03. In physical units, these correspond to RMSE = 0.0470 kJ/m^2^ and MAE = 0.0247 kJ/m^2^ for the full dataset, and RMSE = 0.0194 kJ/m^2^ and MAE = 0.0103 kJ/m^2^ for the fiber-containing subset. These values represent internal sample-wise predictive accuracy within the compiled database.
(3) Source-aware validation showed that cross-source generalization was substantially weaker than sample-wise prediction. GroupKFold validation yielded RMSE = 0.273 kJ/m^2^ and MAE = 0.225 kJ/m^2^, while leave-one-source-out validation yielded RMSE = 0.344 kJ/m^2^ and MAE = 0.311 kJ/m^2^. These results indicate that the reported sample-wise R^2^ values should be interpreted as internal predictive accuracy rather than definitive source-independent generalization.
(4) The correlation matrices and SHAP analyses together indicate that temperature and fiber-related variables play leading roles in controlling fracture energy, whereas volumetric parameters significantly modulate the magnitude and stability of the toughening effect. Strong internal correlations among fiber descriptors also suggest that product-level parameter binding should be considered when interpreting variable importance.
(5) The proposed dosage–response curve cluster representation extends the study beyond point prediction by comparing the background-dependent response of Gf within the observed fiber-dosage range. Rather than indicating a universal monotonic improvement, the bounded scan shows that the dosage effect is nonlinear and strongly conditioned by temperature, volumetric state, loading rate, and fiber descriptors. Because the current literature-derived database contains Fiber% values only up to 0.678%, no validated conclusion was drawn for fiber contents beyond this range.
(6) Overall, the study provides a source-aware and physically interpretable workflow that links prediction, factor identification, and bounded dosage-oriented comparison. Its main value lies in preliminary screening and mechanism-consistent interpretation, while future work should expand the source-balanced database and conduct standardized external experimental validation.

## Figures and Tables

**Table 1:** Definition, unit, encoding method, and physical meaning of input variables.

Variable	Definition	Unit	Encoding	Physical Meaning
OAC	Optimum asphalt content	%	Continuous	Binder content and matrix cohesion
AV	Air voids	%	Continuous	Compaction state and crack-propagation space
Temp	Test temperature	°C	Continuous	Viscoelastic state and brittleness
Rate	Loading rate	mm/min	Continuous	Rate-dependent fracture response
Fiber%	Fiber dosage	%	Continuous	Amount of reinforcing phase
L	Fiber length	mm	Continuous	Bridging length and pull-out path
D	Fiber diameter	μm	Continuous	Aspect ratio and interfacial area
E	Fiber elastic modulus	GPa	Continuous	Stiffness contribution to bridging
TS	Fiber tensile strength	MPa	Continuous	Resistance to rupture during crack opening
Gf	SCB fracture energy	kJ/m^2^	Target	Energy consumed per unit fracture area

**Table 2:** Descriptive statistics of variables in the full dataset.

Parameter	Unit	Mean	Std	Min	25%	50%	75%	Max
OAC	%	5.565	0.449	4.342	5.257	5.561	5.865	6.734
AV	%	5.425	1.329	2.072	4.189	5.367	6.492	9.328
Temp	°C	−11.632	20.347	−42	−24	−18	−10	34
Rate	mm/min	1.969	2.672	0.5	0.5	0.5	1	10
Fiber%	%	0.126	0.181	0	0	0	0.3	0.678
L	mm	4.360	6.844	0	0	0	10.604	31.810
D	μm	12.587	36.133	0	0	0	13.876	243.083
E	GPa	22.902	52.872	0	0	0	9.185	249.205
TS	MPa	621.811	1118.879	0	0	0	726.496	4336.223
Fracture energy (Gf)	kJ/m^2^	0.448	0.258	0.005	0.279	0.374	0.585	1.324

**Table 3:** Hyperparameter settings of the machine learning models.

Machine Learning Model	Parameter 1	Value 1	Parameter 2	Value 2	Parameter 3	Value 3
CatBoost	iterations	60	learning_rate	0.08	depth	5
XGBoost	n_estimators	60	learning_rate	0.08	max_depth	3
GBM	n_estimators	60	learning_rate	0.08	max_depth	3
Extra Trees	n_estimators	40	min_samples_leaf	1	min_samples_split	2
Multi-layer perceptron (MLP)	hidden_layer_sizes	(24, 12)	batch_size	16	max_iter	150

**Table 4:** Five-fold cross-validation R^2^ results of the fiber-containing subset.

Model	Fold 1	Fold 2	Fold 3	Fold 4	Fold 5	Average	Std
CatBoost	0.493	0.251	0.35	−0.164	0.203	0.227	0.245
XGBoost	0.488	0.362	0.342	0.065	0.299	0.311	0.155
GBM	0.316	0.237	0.36	0.108	0.296	0.263	0.097
Extra Trees	0.314	0.314	0.471	−0.232	0.238	0.221	0.267
Multi-layer perceptron (MLP)	−0.537	−1.839	−0.105	−0.628	−0.284	−0.679	0.681

**Table 5:** Source-aware validation diagnosis of the selected model.

Validation Strategy	Grouping Variable	Mean R^2^	RMSE (kJ/m^2^)	MAE (kJ/m^2^)	Interpretation
Sample-wise test	Random sample split	0.890	0.047	0.025	Internal prediction
Fiber-containing subset	Random sample split	0.940	0.019	0.010	Fiber-dominated internal prediction
GroupKFold	SourceID	−0.887	0.273	0.225	Cross-source diagnosis
Group shuffle split	SourceID	−2.110	0.323	0.278	Repeated source-held-out diagnosis
Leave-one-source-out	SourceID	−5.417	0.344	0.311	Strict source-held-out diagnosis

## Data Availability

The original contributions presented in this study are included in the article/Supplementary Materials. Further inquiries can be directed to the corresponding author.

## Supplementary Materials

The following supporting information can be downloaded at: https://www.mdpi.com/article/10.3390/ma19102172/s1, Table S1, The final source-tracked SCB fracture-energy modeling matrix with RecordID and SourceID fields; Table S2, The SourceID mapping and sample-count summary; Code S1, The source-aware validation script; Output S1, The source-aware validation results.

## Abbreviations

SCB	semi-circular bending
Gf	fracture energy
OAC	optimum asphalt content
AV	air voids
Temp	test temperature
Rate	loading rate
L	fiber length
D	fiber diameter
E	elastic modulus
TS	tensile strength
SHAP	SHapley Additive exPlanations
GBM	gradient boosting machine
XGBoost	extreme gradient boosting
MLP	multi-layer perceptron
RMSE	root mean square error
MAE	mean absolute error
LOSO	leave-one-source-out
R2	coefficient of determination
MSE	mean squared error
RecordID	record identifier
SourceID	source identifier
ANN	artificial neural network
GEP	gene expression programming
AFQM	augmented full quadratic model
SVEM	self-validated ensemble modeling
CatBoost	categorical boosting
Extra Trees	extremely randomized trees
APC	article processing charge

## Appendix A. Source-ID Distribution of the Final Modeling Dataset

The final SCB fracture-energy modeling matrix contained 261 records from nine source groups. Table A1 reports the number of records contributed by each SourceID. This table was used to document source-level data balance and to support the source-aware validation diagnosis.

**Table A1:** Number of SCB fracture-energy records in each SourceID group.

SourceID	Number of Records
S5	64
S11	52
S7	48
Marasteanu2007_ROSA38925	39
AlbertaInnovates2021_Hesp	28
S6	16
S8	10
S10	2
S9	2
Total	261
